# Snai1-induced partial epithelial–mesenchymal transition orchestrates p53–p21-mediated G2/M arrest in the progression of renal fibrosis via NF-κB-mediated inflammation

**DOI:** 10.1038/s41419-020-03322-y

**Published:** 2021-01-05

**Authors:** Ruochen Qi, Jiyan Wang, Yamei Jiang, Yue Qiu, Ming Xu, Ruiming Rong, Tongyu Zhu

**Affiliations:** 1 Department of Urology, Zhongshan Hospital, Fudan University, Shanghai, 200032 P. R. China; 2 Shanghai Medical College, Fudan University, Shanghai, 200032 P.R. China; 3 Shanghai Key Laboratory of Organ Transplantation, Shanghai, 200032 P. R. China; 4 Department of Transfusion, Zhongshan Hospital, Fudan University, Shanghai, 200032 P. R. China

**Keywords:** Cell-cycle exit, Mechanisms of disease

## Abstract

Renal fibrosis is the common feature of all progressive kidney diseases and exerts great burden on public health worldwide. The maladaptive repair mechanism of tubular epithelial cells, an important mediator of renal fibrogenesis, manifests with partial epithelial–mesenchymal transition (EMT) and cell cycle arrest. The aim of this study is to investigate the possible correlation between partial EMT and cell cycle arrest, and elucidate the underlying mechanism. We examined human kidney allograft samples with interstitial fibrosis and three mice renal fibrosis models, unilateral ureter obstruction (UUO), ischemia–reperfusion injury, and Adriamycin nephropathy. The partial EMT process and p53–p21 axis were elevated in both human allograft with interstitial fibrosis, as well as three mice renal fibrosis models, and showed a time-dependent increase as fibrosis progressed in the UUO model. Snai1 controlled the partial EMT process, and led to parallel changes in renal fibrosis, G2/M arrest, and inflammation. p53–p21 axis arrested cell cycle at G2/M, and prompted partial EMT and fibrosis together with inflammation. NF-κB inhibitor Bay11-7082 disrupted the reciprocal loop between Snai1-induced partial EMT and p53–p21-mediated G2/M arrest. We demonstrated the reciprocal loop between partial EMT and G2/M arrest of TECs during renal fibrogenesis and revealed NF-κB-mediated inflammatory response as the underlying mechanism. This study suggests that targeting NF-κB might be a plausible therapeutic strategy to disrupt the reciprocal loop between partial EMT and G2/M arrest, therefore alleviating renal fibrosis.

## Introduction

Renal fibrosis is the common feature of all progressive chronic kidney diseases (CKD). Progression of CKD usually leads to the need of renal replacement therapy, either dialysis or kidney transplantation. Currently, CKD affects >10% of the global population, increasing great burden on public health worldwide^1^. Renal fibrosis is characterized with deposition of extracellular matrix (ECM), including collagen fibers and fibronectin, within the kidney tissue along with gradual loss of residual renal function^2^. Development of renal fibrosis involves variety of different cells and signaling pathways, making it difficult to design a specific therapy to counteract or even reverse its progression^3^. Thus, it is of great importance to explore the linkage between these different molecular mechanisms and pathways.

Renal tubular epithelial cells (TECs) are the most abundant cells within the kidney and are responsible for reabsorption, excretion, as well as concentration. For a long time, TECs have been deemed as the innocent victims upon injury, which is partly true, since TECs are always on the frontline facing toxins, hypoxia, etc. However, recent findings demonstrate that TECs are actually essential participants in the development of renal fibrosis^4,5^. TECs start a series of intracellular changes and intercellular signaling upon assault, struggle to make up for the losses, and restore kidney function. However, if the injury becomes chronic and persistent, the repair mechanisms become unbalanced and turn TECs to a pro-fibrotic phenotype^4^. This process is called the maladaptive repair mechanism. These findings evoked targeting the maladaptive repair mechanism as a strategy to prevent renal fibrosis.

Epithelial–mesenchymal transition (EMT) is the indispensable process during embryonic development, cancer metastasis, and organ fibrosis^6^. Its implication in renal fibrosis has been discussed for nearly two decades, and till now, reach the agreement that partial EMT, loss of some epithelial features meanwhile acquiring some mesenchymal features, is a manifestation of maladaptive repair. Partial EMT enables TECs with elevated migration capacity, resistance to apoptosis, ability to produce inflammatory cytokines, and even direct production of ECM^7,8^. Several studies have shown that zinc finger protein Snai1 is the main regulator of the partial EMT process during renal fibrosis^9,10^.

Cell cycle arrest manifests another form of maladaptive repair of TECs and has been found in correlation with the development of renal fibrosis in a variety of animal models, including aristolochic acid-induced kidney injury^11^, cisplatin-induced injury^12^, and ischemia–reperfusion injury (IRI)^13,14^. Cell cycle arrest, especially G2/M arrest are mostly mediated by p53, p21, and p16 (refs. ^15,16^). Study have shown that specific ablation of p53 in proximal TECs alleviates IRI, indicating the importance of p53–p21 axis in kidney injury. Arrested TECs become an important source of pro-inflammatory and pro-fibrotic cytokines, which is known as the senescence-associated secretory phenotype (SASP)^1,17^. Given the evidence that partial EMT and cell cycle arrest are both part of the mechanism of maladaptive repair of TECs, and both are strongly associated with inflammation, there is a reason to hypothesize that the partial EMT process could mutually orchestrate cell cycle arrest of TECs via inflammation and push forward the progress of renal fibrosis.

In this study, we examined the coexistence of partial EMT and p53–p21-mediated cell cycle arrest in human kidney samples and three renal fibrosis animal models. We further demonstrated the reciprocal loop between partial EMT and cell cycle arrest, and explored the underlying mechanism. Our results suggest that Snai1-induced partial EMT mutually orchestrates with p53–p21-mediated cell cycle arrest in TECs via NF-κB pathway, and together, push forward the progression of renal fibrosis.

## Results

### Partial EMT and p53–p21 axis are induced in kidney allograft with chronic rejection

**Fig. 1:**
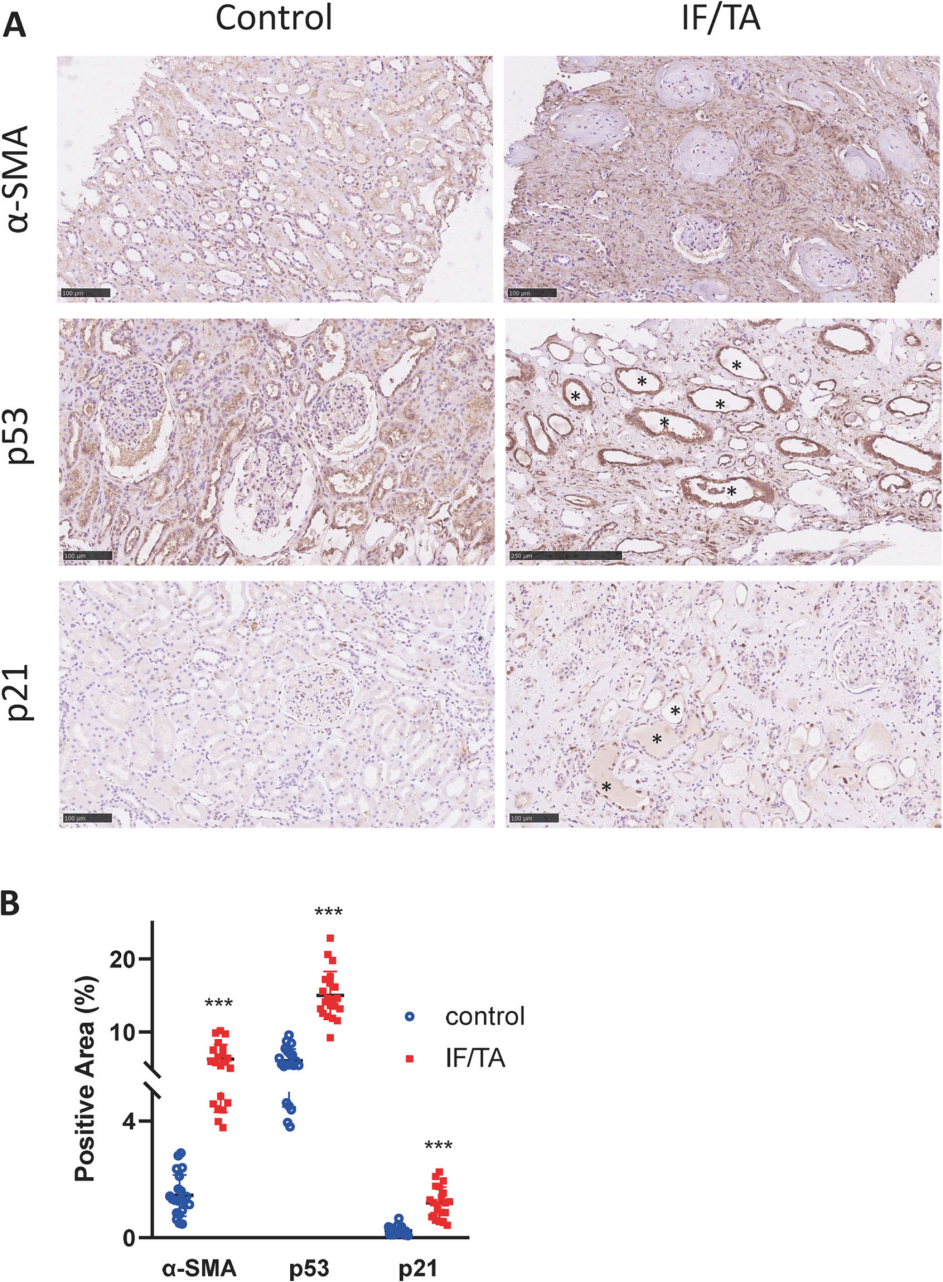
α-SMA and p53–p21 axis are upregulated in kidney allograft with chronic rejection. **A** Immunohistochemistry staining of α-SMA, p53, and p21 in allograft kidneys and **B** their quantifications. Three random fields were chosen in each sample. Scale bar = 100 μm. ***P < 0.001.

Kidney allograft with chronic rejection is characterized with interstitial fibrosis and tubular atrophy^18^. To examine whether partial EMT and p53–p21 axis are activated in kidney transplant recipients with chronic rejection, we collected biopsy samples with pathological proven IF/TA, as well as healthy kidney as controls. Immunohistochemistry staining of the EMT marker α-SMA revealed abundant expression in the interstitial area in allografts with IF/TA (Fig. 1A). P53 and p21 were also upregulated in these kidneys, both stained within the dilated tubular cells (Fig. 1A, lumen labeled with *). Semiquantitative analysis of the immunohistochemistry staining revealed the upregulation of these proteins in allografts with IF/TA to be statistically significant (Fig. 1B).

Creatinine level and eGFR level at the time of biopsy were also analyzed. Pearson correlation analysis showed a positive correlation of creatinine level with α-SMA, p53, and p21 expression in allograft biopsies (Fig. S1A–C). In contrast, negative correlation was seen between eGFR level and the expression of these proteins (Fig. S1D–F).

### Partial EMT and p53–p21 axis are induced in animal models with renal fibrosis

To examine whether partial EMT and p53–p21 axis are also induced in animal models, we conducted three frequently used renal fibrosis models, unilateral ureter obstruction (UUO)^19^, IRI^14^, and Adriamycin (ADR) nephropathy induced fibrosis^20^.

**Fig. 2:**
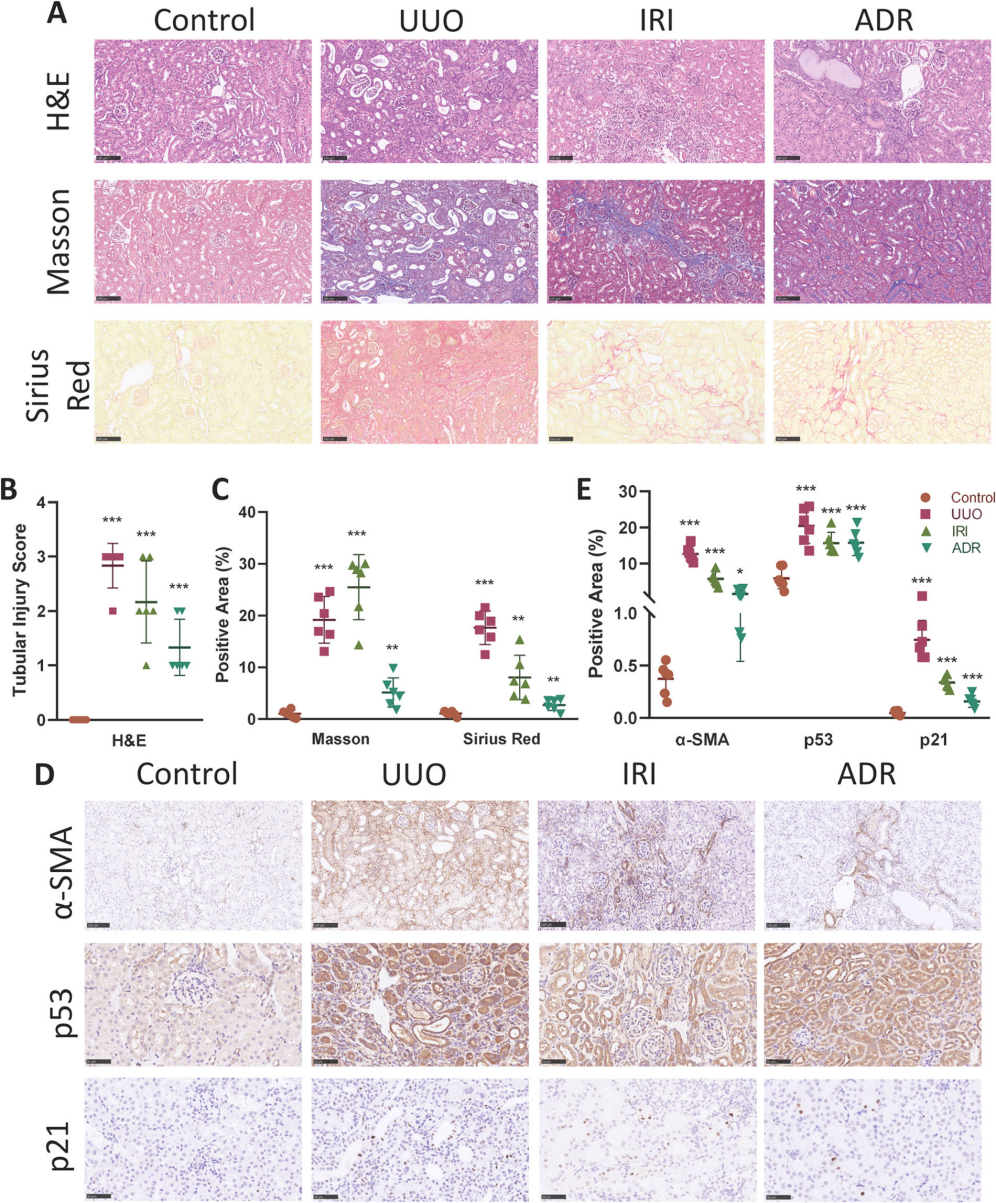
α-SMA and p53–p21 axis are upregulated in UUO, IRI, and ADR nephropathy models. **A** H&E, Masson trichrome, and Sirius red staining of control, UUO, IRI, and ADR nephropathy kidneys. Scale bar = 100 μm. **B** Quantitative tubular injury scores of different groups assessed according to H&E staining. **C** Quantification of Masson trichrome and Sirius staining in different groups. **D** Immunohistochemistry staining of α-SMA, p53, and p21 in different groups and **E** their quantifications. Scale bar = 100 μm for α-SMA and scale bar = 50 μm for p53 and p21. *P < 0.05; **P < 0.01; ***P < 0.001.

In the UUO model, H&E staining revealed dilated tubular lumen with casts, inflammatory cells infiltration, as well as interstitial fibrosis (Fig. 2A, B). Masson trichrome and Sirius red staining demonstrated sufficient deposition of ECM in the interstitium (Fig. 2A, C), indicating renal fibrosis. Immunohistochemistry revealed an induction of α-SMA in the UUO model (Fig. 2D, E), which was confirmed by western blot analysis (Fig. S2A, B). Western blot using kidney tissue lysates also demonstrated the loss of E-cadherin, the epithelial marker of TEC along with the upregulation of Snai1, an important transcription factor of the partial EMT process (Fig. S2A, B). Moreover, the induction of the p53–p21 axis were also proved in the UUO model using immunohistochemistry (Fig. 2D, E), as well as western blot (Fig. S2A, B).

In the IRI and ADR model, renal fibrosis was also successfully induced, demonstrated by H&E staining and staining of ECM (Fig. 2A–C). Immunohistochemistry showed upregulation of α-SMA, p53, as well as p21 compared with control (Fig. 2D, E). Western blot also revealed significant increase of fibronectin, α-SMA, Snail, p53, p21 along with loss of E-cadherin in these two fibrosis animal models compared with control (Fig. S2C–F).

Collectively, these data indicate that the partial EMT process, as well as activation of p53–p21 axis are the common features of development of renal fibrosis, despite the initial injury.

### Partial EMT and p53–p21 axis are upregulated in a time-dependent manner in UUO model

**Fig. 3:**
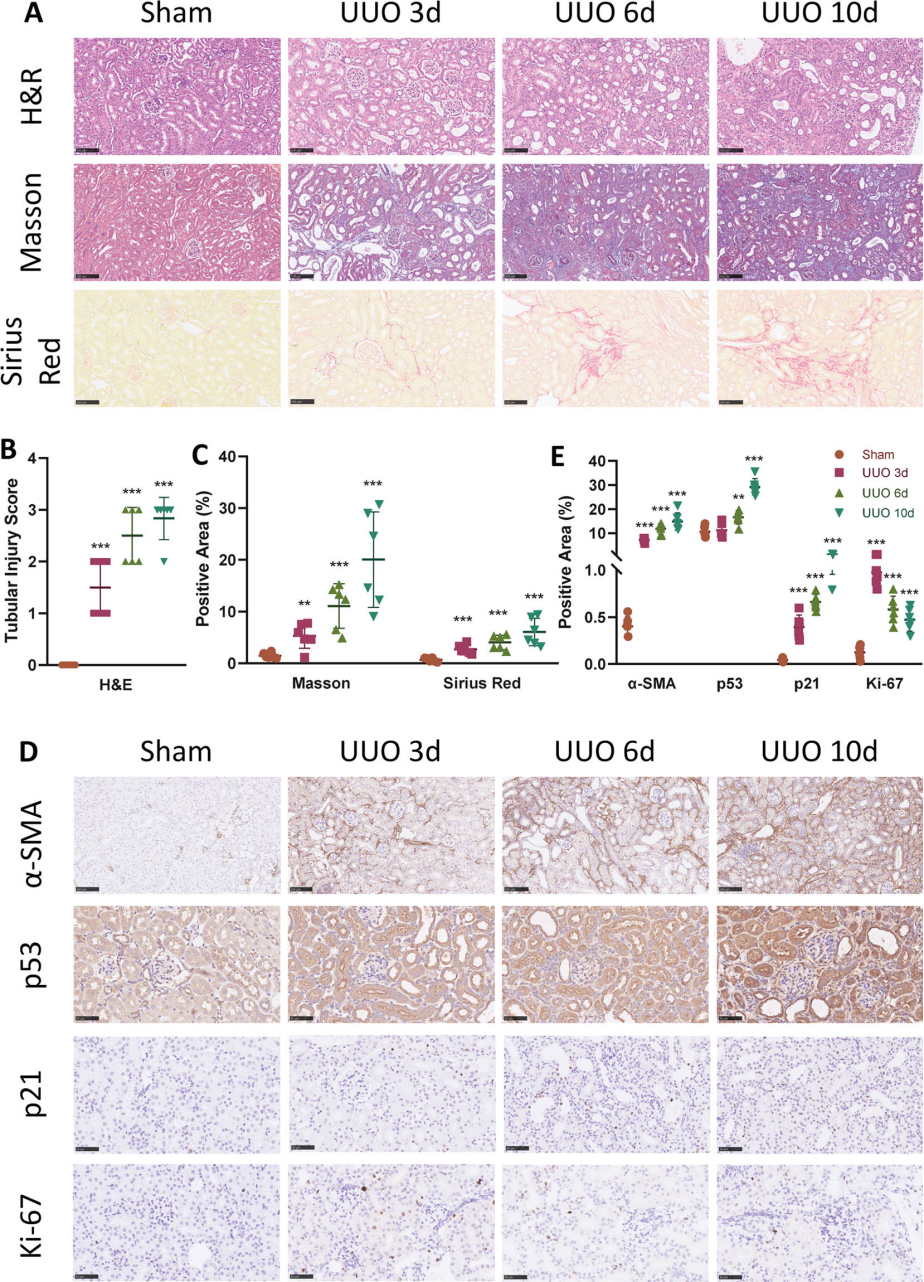
EMT and p53–p21-mediated cell cycle arrest progresses in a time-dependent manner in UUO model. **A** H&E, Masson trichrome, and Sirius red staining of sham, UUO at 3, 6, and 10 days. Scale bar = 100 μm. **B** Quantitative tubular injury scores of different groups assessed according to H&E staining. **C** Quantification of Masson trichrome and Sirius staining in different groups. **D** Immunohistochemistry staining of α-SMA, p53, p21, and Ki-67 in different groups and **E** their quantifications. Scale bar = 100 μm for α-SMA and scale bar = 50 μm for p53, p21, and Ki-67. **P < 0.01; ***P < 0.001 compared with sham group.

To further demonstrate the correlation of partial EMT and p53–p21 axis with renal fibrosis, we conducted the UUO-induced renal fibrosis model and chose three different time points to sacrifice the mice. A gradual increase in severity of tubular injury and interstitial fibrosis was observed from 3 to 6 days and eventually 10 days after ureter obstruction, shown by H&E staining and tubular injury scoring (Fig. 3A, B). Increase of ECM deposition was also observed in a time-dependent manner, demonstrated by Masson trichrome, Sirius red staining (Fig. 3A, C), as well as fibronectin expression (Fig. S3A, B).

Extent of the partial EMT progress underwent a gradual increase in accordance with the severity of renal fibrosis. Immunohistochemistry staining revealed elevated expression of α-SMA as fibrosis progressed (Fig. 3D, E). Western blot analysis showed consistent results, with increase of α-SMA and Snai1, along with decrease of E-cadherin in a time-dependent manner (Fig. S3A, B).

Expression of p53 and p21 also presented a similar pattern, shown by immunohistochemistry and western blot (Figs. 3D, E and S3A, B). Since p53–p21 axis mediates cell cycle arrest, we also examined the expression of Ki-67, a cell proliferation marker^21^. Three days after ureter ligation, Ki-67 expression was significantly increased in TEC nucleus, indicating active proliferation of TEC to remedy the loss caused by injury (Fig. 3D, E). However, as the ligation persisted, a gradual decreased Ki-67 expression was seen at 6 and 10 days after ligation, compared with 3 days. This is in accordance with the increased activity of the p53–p21 axis and the arrested cell cycle. Collectively, these results indicate that the partial EMT and p53–p21 axis were elevated in a time-dependent manner in the UUO-induced renal fibrosis model.

### Snai1-induced partial EMT regulates cell cycle arrest and renal fibrosis

**Fig. 4:**
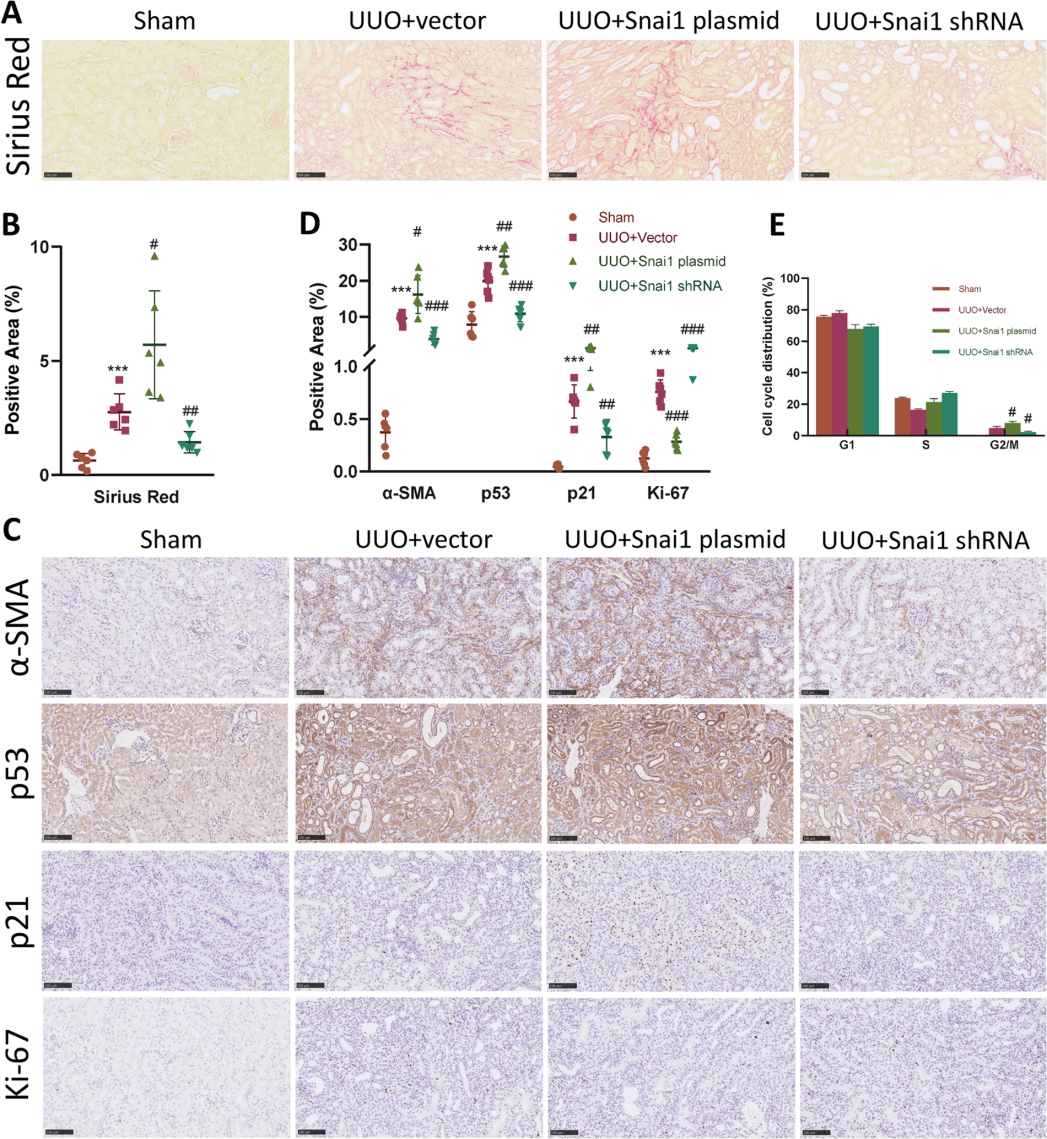
Regulation of Snai1 expression changes EMT and G2/M arrest. UUO mice were injected with plasmid encoding either Snai1 mRNA or shRNA to regulate the expression of Snai1 1 day after UUO establishment. **A** Sirius red staining of kidney from sham, UUO + vector, UUO + Snai1 plasmid, and UUO + Snai1 shRNA group, and **B** their quantifications. Scale bar = 100 μm. **C** Immunohistochemistry staining of α-SMA, p53, p21, and Ki-67 in different groups, and **D** their quantifications. Scale bar = 100 μm for α-SMA and scale bar = 50 μm for p53, p21, and Ki-67. **E** Cell cycle distribution of TECs isolated from obstructed kidneys in different groups. ***P < 0.001 compared with the sham group. ^#^*P* < 0.05; ^##^*P* < 0.01; ^###^*P* < 0.001 compared with the UUO + vector group.

To further investigate the correlation between partial EMT and cell cycle arrest. We designed two plasmids either encoding Snai1 protein or Snai1 shRNA to regulate the expression of Snai1, therefore control the partial EMT process in the established UUO model^9^. Plasmid was injected through the tail vein in a hydrodynamic gene delivery manner^22^ 1 day after the ureter was ligated. Sirius red and Masson trichrome staining showed that upregulation of Snai1 by plasmid injection caused an increased deposition of ECM in the interstitium, while downregulation of Snai1 attenuated it (Figs. 4A, B and S4A, B). Protein expression level of collagen IV and collagen I confirmed this result (Fig. S4C, D).

The partial EMT process, marked as expression of α-SMA, was significantly upregulated in the Snai1 plasmid injection group, while was ameliorated in the Snai1 shRNA group (Fig. 4C, D). Western blot analysis revealed consistent results, with changes of E-cadherin and Snai1 level (Fig. S4C, D).

Cell cycle arrest changed along with the regulation of Snai1. Expression of p53 and p21 were also increased in the Snai1 plasmid group, with decreased Ki-67 expression (Figs. 4C, D and S4C, D). Notably, p53 and p21 were mainly expressed in the TECs, especially the dilated ones. To further explore the cell cycle arrest process, we isolated TECs from obstructed kidneys and applied flow cytometry to analyze the cell cycle distribution. Ligation of the kidney caused an increase of cells arrested at G2/M phase, which was further aggravated in the Snai1 plasmid group. The cells arrested partially decreased with the downregulation of Snai1 (Fig. 4E). This corresponded with the expression level of the p53–p21 axis in the obstructed kidney.

Taken together, these data indicate that Snai1 not only regulates the partial EMT process, but also induces a G2/M arrest mediated by p53–p21 axis in UUO model.

### Snai1-induced partial EMT regulates inflammation in the interstitium

**Fig. 5:**
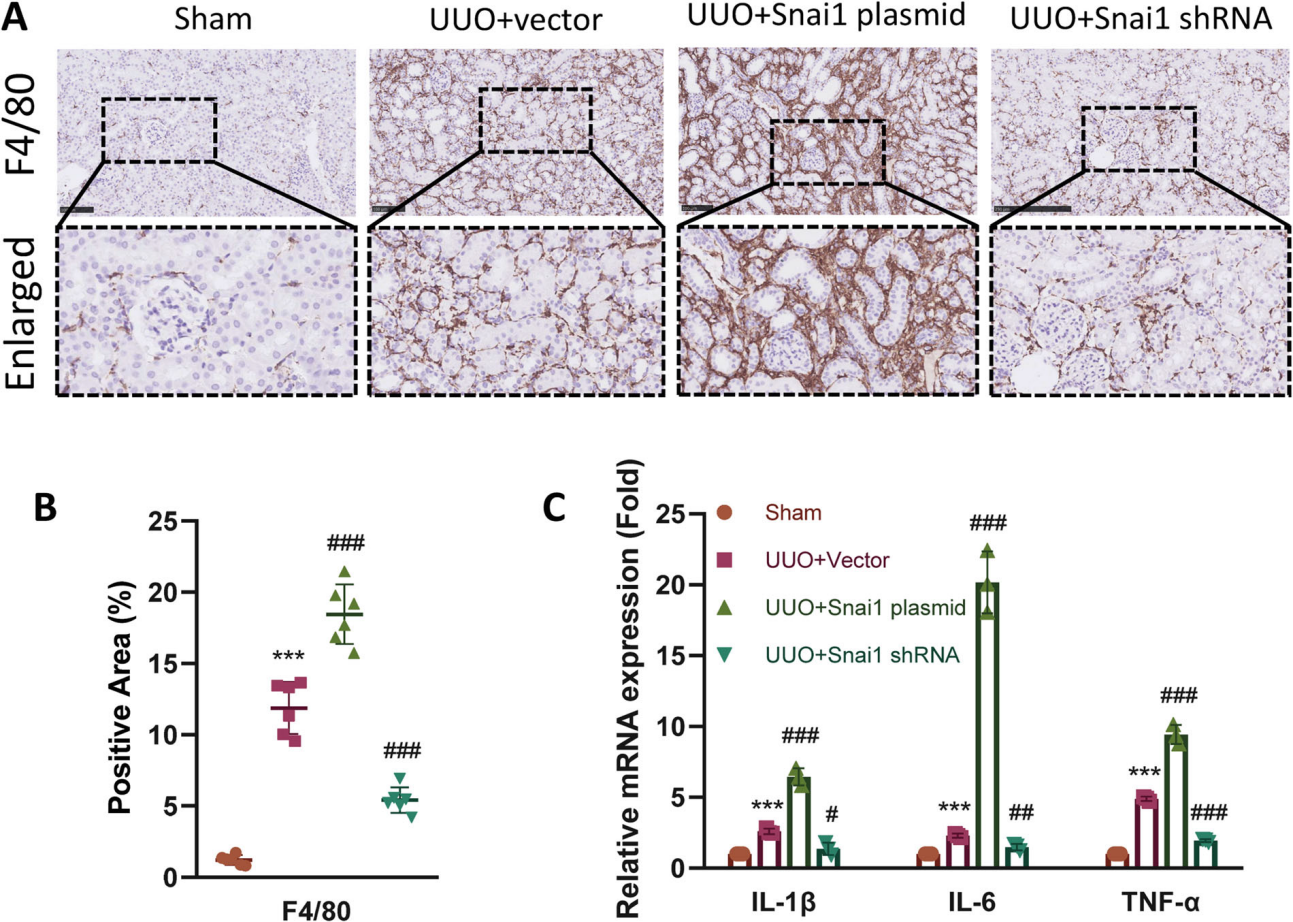
Regulation of Snai1 expression changes renal inflammation. **A** Immunohistochemistry staining and enlarged images of F4/80-positive macrophages in the interstitium of sham, UUO + vector, UUO + Snai1 plasmid, and UUO + Snai1 shRNA group and **B** their quantifications. Scale bar = 100 μm. **C** Relative mRNA expression of IL-1β, IL-6, and TNF-α in kidneys from different groups. ***P < 0.001 compared with the sham group. ^#^*P* < 0.05; ^##^*P* < 0.01; ^###^*P* < 0.001 compared with the UUO + vector group.

Inflammation is an important mediator of renal fibrosis^15^, and sustained inflammation contribute to the maladaptive repair of injured TECs^4^. Therefore, we hypothesized that the regulation of Snai1 might also interact with inflammatory response. To elucidate this, we first applied immunohistochemistry of F4/80, the macrophage marker. UUO led to infiltration of macrophages in the interstitium, which was aggravated in the Snai1 plasmid group. Downregulation of Snai1 by shRNA attenuated this phenomenon (Fig. 5A, B). Next, we investigated the expression of several inflammatory cytokines, IL-1β, IL-6, and TNF-α. These factors are also important constituent of SASP. Consistent with F4/80, all three cytokines were upregulated after Snai1 overexpression and decreased with Snail1 knockdown compared with the vector group (Fig. 5C). Thus, these results indicate that Snai1-induced partial EMT process also participates in the inflammatory response in the interstitium.

### p53–p21 axis-induced cell cycle arrest regulates partial EMT and renal fibrosis

**Fig. 6:**
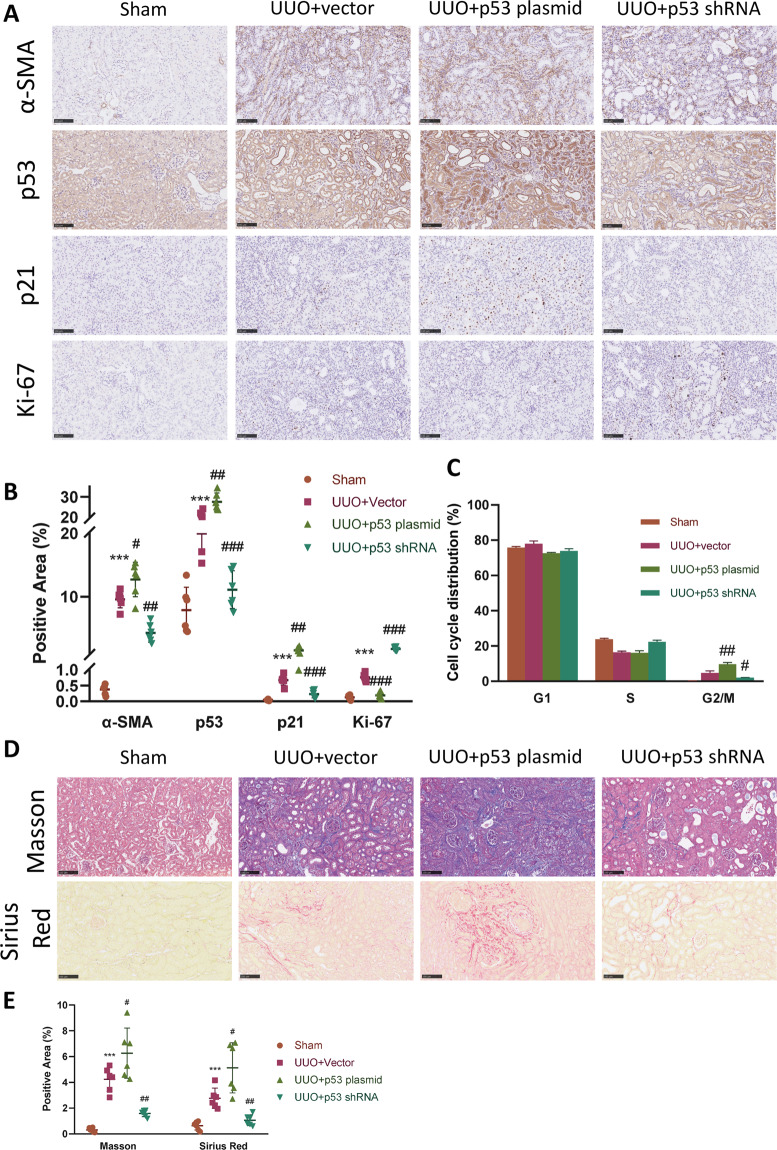
Regulation of p53 expression changes G2/M arrest and EMT. To regulate the expression of the p53–p21 axis, UUO mice were injected with plasmid encoding either p53 mRNA or p53 shRNA 1 day after UUO establishment. **A** Immunohistochemistry staining of α-SMA, p53, p21, and Ki-67 in kidneys of sham, UUO + vector, UUO + p53 plasmid, and UUO + p53 shRNA group and **B** their quantifications. Scale bar = 100 μm for α-SMA and scale bar = 50 μm for p53, p21, and Ki-67. **C** Cell cycle distribution of TECs isolated from obstructed kidneys in different groups. **D** Masson trichrome and Sirius red staining, and **E** their quantifications in different groups. Scale bar = 100 μm. ***P < 0.001 compared with the sham group. ^#^*P* < 0.05; ^##^*P* < 0.01; ^###^*P* < 0.001 compared with the UUO + vector group.

Given the results above, we next wondered whether regulation of the p53–p21 axis could also exert some effects on the partial EMT process, as well as renal fibrosis. Therefore, we designed plasmid encoding p53 protein, as well as plasmid encoding p53 shRNA. Similarly, plasmid was injected 1 day after UUO was established. Overexpression and downregulation of p53 was confirmed with immunohistochemistry, as well as western blot (Figs. 6A, B and S5A, B). P21 also changed accordingly under the regulation of p53, with Ki-67 changed in an opposite manner (Figs. 6A, B and S5A, B). We then analyzed cell cycle distribution to determine whether changes of p53–p21 axis affected cell cycle. Not surprisingly, upregulation of p53 led to substantial TECs arrested at G2/M, while downregulation of p53 mitigated this (Fig. 6C).

Expression of the EMT marker α-SMA changed in parallel with p53, demonstrated by immunohistochemistry and western blot (Figs. 6A, B and S5A, B). Acquisition of Snai1 and loss of E-cadherin also changed in response to p53 regulation (Fig. S5A, B). Finally, extend of renal fibrosis was aggravated with p53 overexpression, demonstrated by Masson trichrome, Sirius red staining (Fig. 6D, E), as well as expression of ECM proteins (Fig. S5A, B). These data demonstrate that changes in p53–p21 axis-mediated cell cycle arrest could also modulate the partial EMT process, as well as renal fibrosis.

### p53–p21 axis-induced cell cycle arrest regulates inflammation in the interstitium

**Fig. 7:**
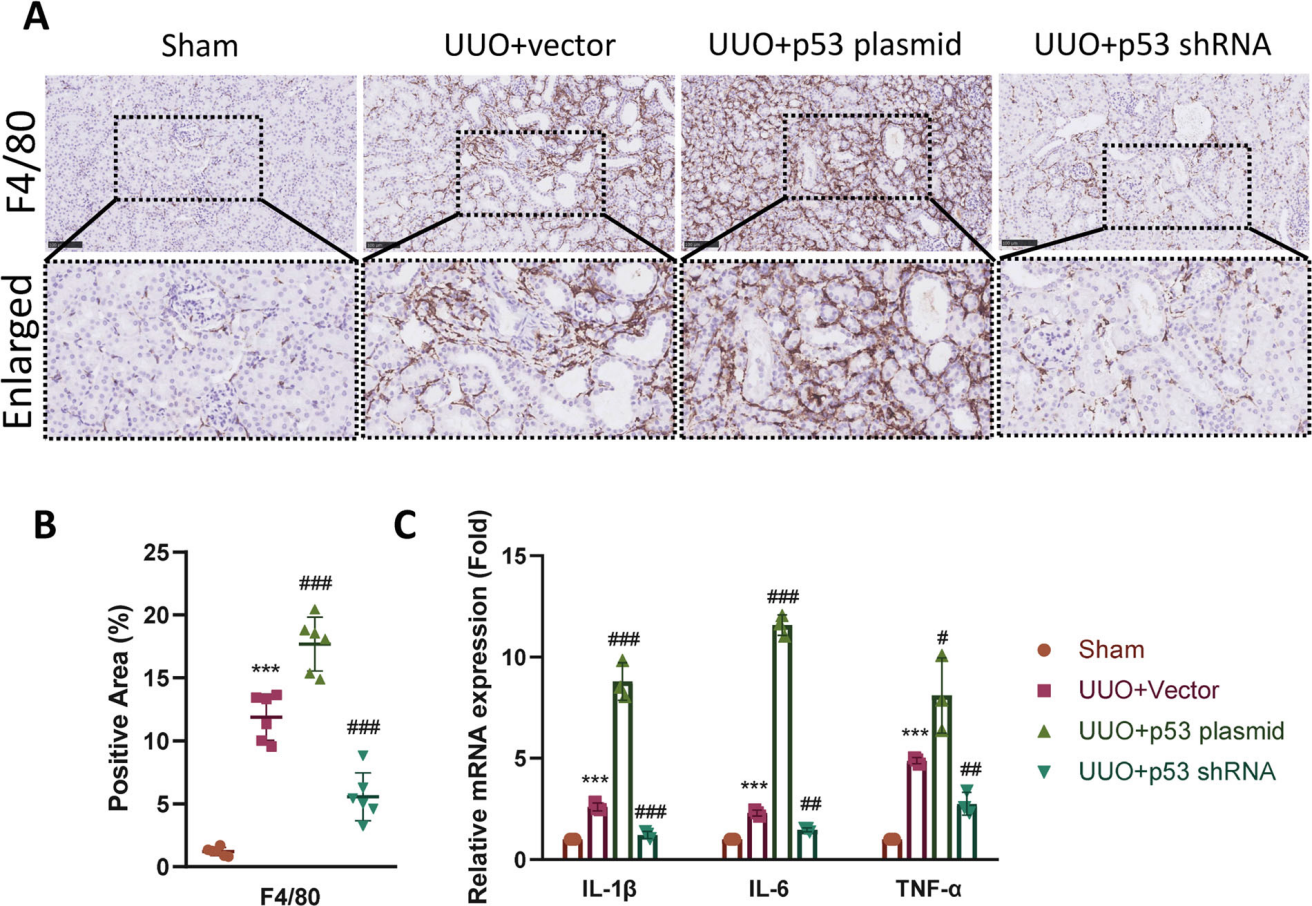
Regulation of p53 expression changes renal inflammation. **A** Immunohistochemistry staining and enlarged images of F4/80-positive macrophages in the interstitium of sham, UUO + vector, UUO + p53 plasmid, and UUO + p53 shRNA group and **B** their quantifications. Scale bar = 100 μm. **C** Relative mRNA expression of IL-1β, IL-6, and TNF-α in kidneys from different groups. ***P < 0.001 compared with the sham group. ^#^*P* < 0.05; ^##^*P* < 0.01; ^###^*P* < 0.001 compared with the UUO + vector group.

Cells arrested at G2/M phase tend to secrete inflammatory cytokines to recruit immune cells, this process is known as the SASP^1^. Therefore, we decided to determine whether the regulation of p53 also led to changes in the inflammatory response. F4/80 was detected using immunohistochemistry. Overexpression of p53 led to significant increase in F4/80-positive macrophages infiltration in the interstium compared with the vector group, while knockdown of p53 ameliorated the infiltration (Fig. 7A, B). We then determined the mRNA expression level of IL-1β, IL-6, and TNF-α using quantitative PCR. Results showed that injection of p53 plasmid caused a further increase of these cytokines compared with UUO + vector group, while downregulation of p53 decreased the inflammatory response (Fig. 7A, B). Taken together, these results suggest that the p53–p21 axis also takes a part in the regulation of interstitial inflammation in the fibrotic kidney.

### Snai1-induced partial EMT orchestrates p53–p21 axis via NF-κB pathway

Given the results above, there is a reason to draw the conclusion that Snai1-induced partial EMT form a positive regulation loop together with p53–p21 axis-mediated cell cycle arrest. Next, we decided to investigate the underlying mechanism regulating this reciprocal communication. NF-κB is one of the key regulators of inflammation and several studies have indicated its role in renal fibrosis^23–25^. Our previous data has already revealed the correlation between partial EMT and cell cycle arrest with inflammation. Thus, we hypothesized that NF-κB might be the key linkage molecule between Snai1-induced partial EMT and p53–p21 axis-mediated cell cycle arrest. To prove this, we first injected either Snai1 plasmid or p53 plasmid to mice underwent UUO, and treated half of them with Bay11-7082, a NF-κB pathway inhibitor. Treatment of Bay11-7082 significantly attenuated the ECM deposition in both Snai1 and p53 overexpression group, shown by Masson trichrome and Sirius red staining (Fig. S6A, B).

**Fig. 8:**
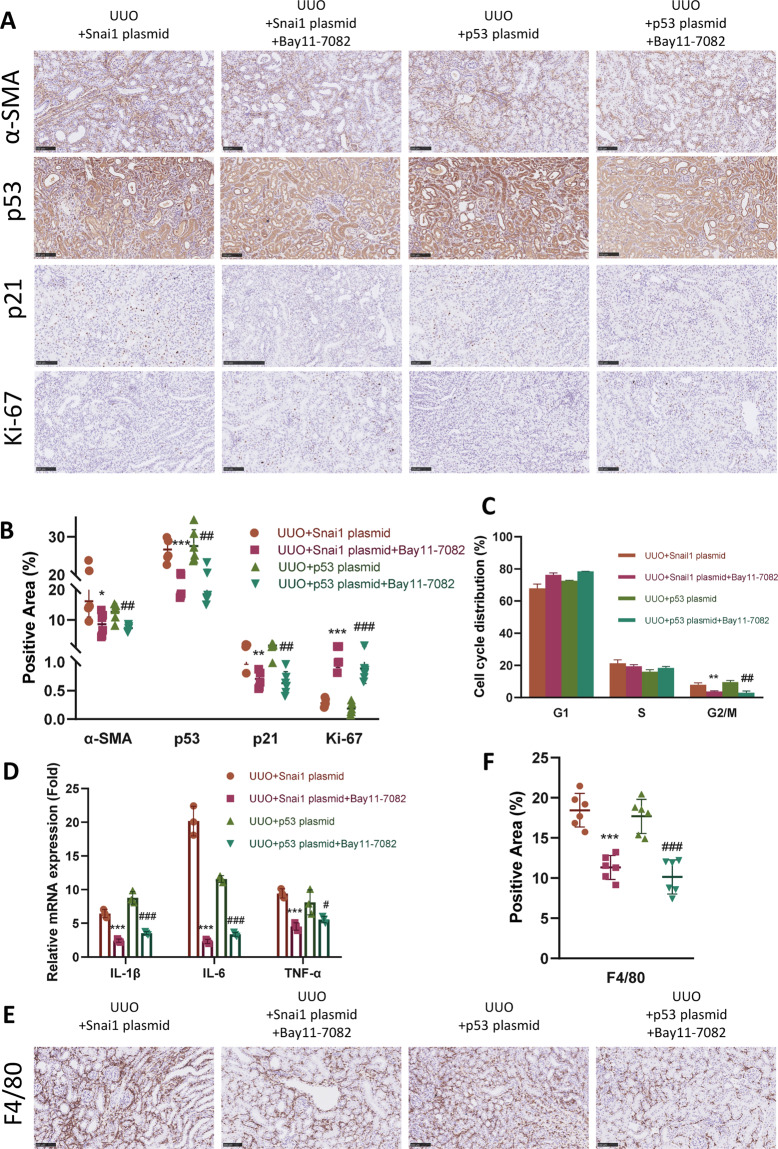
Snai1-induced EMT orchestrates p53–p21 axis via NF-κB pathway. UUO mice were injected with Bay11-7082, the NF-κB inhibitor after the administration of either Snai1 or p53 plasmid. **A** Immunohistochemistry staining of α-SMA, p53, p21, and Ki-67 in different groups and **B** their quantifications. Scale bar = 100 μm for α-SMA and scale bar = 50 μm for p53, p21, and Ki-67. **C** Cell cycle distribution of TECs isolated from obstructed kidneys in different groups. **D** Relative mRNA expression of IL-1β, IL-6, and TNF-α in kidneys from different groups. **E** Immunohistochemistry staining of F4/80 in different groups and **F** their quantifications. Scale bar = 100 μm. *P < 0.05; **P < 0.01; ***P < 0.001 compared with UUO + Snai1 plasmid group. ^#^*P* < 0.05; ^##^*P* < 0.01; ^###^*P* < 0.001 compared with UUO + p53 plasmid group.

Bay11-7082 attenuated the α-SMA level in the Snai1 overexpression group (Fig. 8A, B), indicating amelioration of the partial EMT process. Moreover, this NF-κB inhibitor also mitigated the cell cycle arrest in this group, shown by decreased expression of p53–p21 axis, regained Ki-67 level (Fig. 8A, B), as well as fewer TECs arrested at G2/M phase (Fig. 8C).

In the p53 plasmid group, Bay11-7082 also showed its effect. Expression level of α-SMA, p53, and p21 were all downregulated after Bay11-7082 treatment, together with increased Ki-67 level (Fig. 8A, B). Cell arrested at G2/M was also decreased, indicating cells going back to proliferation (Fig. 8C).

Furthermore, Bay11-7082 treatment downregulated the inflammatory response in both groups, with less inflammatory cytokines expression (Fig. 8D) and fewer F4/80-positive macrophages infiltration (Fig. 8E, F). Taken together, these data demonstrate that NF-κB-mediated inflammatory response is the bond that link the reciprocal loop between Snai1-induced partial EMT and p53–p21 axis-mediated cell cycle arrest.

## Discussion

In the current study, we identified the activation of the partial EMT process and the p53–p21 axis in kidney allograft samples with IF/TA, as well as three different renal fibrosis animal models, including UUO, IRI, and ADR nephropathy-induced renal fibrosis. We further showed that partial EMT and cell cycle arrest aggravated in a time-dependent manner in a UUO model along with the progression of fibrosis. We then demonstrated that the regulation of Snai1 in UUO model not only changed the EMT process, but also led to changes in cell cycle arrest, interstitial inflammation, and renal fibrosis in parallel. Changes in p53–p21-mediated cell cycle arrest in UUO model also caused parallel changes in EMT and inflammation. We further elucidated that the reciprocal loop between partial EMT and cell cycle arrest was bonded by NF-κB-mediated inflammation (Fig. S7). These findings suggest that inhibition of NF-κB-mediated inflammation might be a plausible therapeutic target to disrupt the interaction between partial EMT and cell cycle arrest, therefore alleviate renal fibrosis.

As our understanding toward the mechanisms of renal fibrosis getting increasingly profound, most of the studies have reached the consensus that the maladaptive repair mechanism of TECs plays an important role in the development of renal fibrosis^4,5^. Multiple studies have pointed out the importance of partial EMT and cell cycle arrest during this process^8,11,26^. However, most of the them deemed partial EMT and cell cycle arrest as two separate biological process, and failed to investigate the underlying contact. To prove that the correlation between partial EMT and p53–p21 axis-mediated cell cycle arrest is a universal phenomenon instead of special cases during fibrogenesis, we conducted three different mice renal fibrosis models with distinct initial injury. UUO induced fibrosis through postrenal obstruction^19^, IRI through hypoxia and reperfusion injury^16^, while ADR through chronic proteinuria^20^. All three models showed that EMT, as well as the p53–p21 axis were activated after injury, indicating that these two processes are common features for the development of renal fibrosis.

EMT is indispensable for embryonic development, recent findings have also shed light on its role in organ fibrosis. Since the first time Iwano et al. found the contribution of EMT to myofibroblast in renal fibrogenesis^7^, there had been a heated debate toward the existence of EMT in renal fibrosis and exactly what percentage of myofibroblast were derived from TECs^6,8,27^. Recent finding has indicated zinc finger protein Snai1 as a main transcription factor controlling the partial EMT process^9^. In our study, we showed that regulating the expression of Snai1 by plasmid caused a parallel change in the α-SMA staining in the interstitium, which was in accordance with previous studies^9,28^. We further found enhanced partial EMT process led to the upregulation of p53–p21 axis expression and aggravated G2/M cell cycle arrest. Though previous study has shown that Snai1-induced partial EMT could lead to G2 arrest in TECs^10^, it is the first time that we report the involvement of p53 in this regulation process.

The progression of partial EMT is in strong association with inflammation. Chronic inflammation is the main initiator of partial EMT and starts a series of intracellular signaling pathways in TECs, including TGF-β, Wnt, Notch, etc.^29^. These pathways help TECs to reprogram and adapt this inflammatory environment. Thus, the partial EMT can be deemed as a kind of adaptation to survive and resist imminent death^27^. TECs underwent partial EMT could also secrete inflammatory cytokines to sustain inflammation, intending to recruit inflammatory cells, and resolve injury^4^. In this study, we demonstrated that the upregulation of partial EMT by Snai1 led to the substantial infiltration of F4/80-positive macrophages and secretion of inflammatory cytokines. Several studies have revealed that targeting NF-κB-mediated inflammation could alleviate partial EMT in renal fibrosis^24,25,30^. Here, we showed that Bay11-7082, the NF-κB inhibitor, could alleviate the partial EMT and renal fibrosis induced by Snai1 upregulation, as well as resolve the inflammatory response in the interstitium.

Cell cycle arrest at G2/M has been observed in a variety of renal fibrosis models^11,31,32^ and has been found to directly regulate renal fibrosis^33^. Cell cycle arrest could be induced by several mediators, including p53, p21, and p16 (refs. ^15,34,35^). In this study, we focused on the expression and effect of the p53–p21 axis. Upregulation of this axis was observed in kidney allografts with IF/TA together with three renal fibrosis animal models in this study. Regulating the expression of this axis also caused corresponding G2/M arrest, changes in partial EMT, and inflammatory response. Cell cycle arrest is a self-regulatory mechanism to avoid inaccurate replication with DNA damage. However, prolonged injury leads to sustained cell cycle arrest and inflammatory cytokines secretion, known as SASP^36^. Several studies have indicated that limiting renal inflammation could rescue TECs from G2/M arrest^23,37^. The present study also confirmed that blocking the NF-κB inflammatory pathway could effectively counteract the G2/M arrest induced by p53 overexpression in UUO model.

Though we focused on the cell cycle arrest regulated by p53–p21 axis in the present study, we have to admit that p53 is involved in other aspects in renal fibrogenesis. Besides cell cycle, p53 also regulates apoptosis and autophagy in TECs^38,39^. Earlier study has shown that p53 drives apoptosis in TECs treated with cisplatin and could be inhibited by Pifithrin-α^12^. Another study showed that proapoptotic p53 targets were reduced in ischemic kidney with p53 deficiency^40^. Other studies have also shed light on the interaction of p53 with TGF-β1/SMAD pathway^41,42^ or noncoding RNA^43,44^. In our study, we concentrated on the correlation between p53–p21 axis and G2/M arrest, and showed positive results; however, p53 is involved in multiple molecular pathways and the exact function of p53 in renal fibrogenesis still needs further exploration.

The current study demonstrated the reciprocal loop between partial EMT and G2/M arrest via NF-κB pathway. Previous studies have also provided some support for this orchestration in another manner. TGF-β1 is the master regulator of renal fibrosis and is in strong association with Snai1 and the partial EMT process^45,46^. Recombinant TGF-β1 is the most effective effector to induce partial EMT in TECs^19,28^. Meanwhile, TGF-β1 is also found to interact with p53 by promoting assembly of the p53–SMAD3 complex^41,47,48^, inhibition of p53 could lead to the blockage of normal TGF-β1 signal transduction^49^. Thus, it is possible that activation of TGF-β1 upon injury starts both the Snai1-induced partial EMT and p53-mediated G2/M arrest. As injury persists, these two processes form a positive loop to orchestrate each other via inflammation. Another shortcoming of this study is that we evaluated the extent of partial EMT, as well as G2/M arrest both in the whole TECs population. However, we failed to observe the same TEC undergoing both processes at the same time. Whether partial EMT and cell cycle arrest could occur in the same TEC during the same period still needs further investigation.

In summary, we showed that partial EMT and p53–p21 axis were upregulated in human kidney allograft with IF/TA, as well as three mice renal fibrosis models. We further demonstrated that the partial EMT, as well as p53–p21-mediated cell cycle arrest progresses in a time-dependent manner during fibrogenesis in a UUO model. We then showed the reciprocal loop between partial EMT and G2/M arrest by regulating the expression of either Snai1 or p53. Finally, we elucidated that this positive loop was mediated by NF-κB-mediated inflammation. Our results indicated that targeting NF-κB might be a plausible therapeutic strategy to disrupt the reciprocal loop between partial EMT and G2/M arrest, therefore alleviating renal fibrosis.

## Materials and methods

### Human kidney biopsy samples

Human kidney biopsy samples were acquired from kidney transplant recipients from Zhongshan Hospital, Fudan University. The IF/TA group consisted of seven patients admitted to the hospital for elevated creatinine level. Kidney biopsy proved them to be IF/TA according to Banff Criteria^18^. The control group consisted of seven kidney recipients with stable creatinine level and protocol biopsy proved healthy allograft. Collection of kidney biopsy and clinical data has gained informed consent and was conducted under the supervision of Ethical Committee of Zhongshan Hospital, Fudan University.

### Animal models and treatment

All animal experiments were conducted under the Guidelines of the Care and Use of Laboratory Animals of Fudan University. All practices have been approved by the Animal Ethical Committee of Zhongshan Hospital, Fudan University. Male C57BL/6 mice and BALB/c mice weighing 22–25 g were purchased from Slac, Inc (Shanghai, China) and were bred in a SPF-grade animal room. For each group, six mice were included. Sample size was determined according to previous experiments. C57BL/6 mice were randomized to the sham group, UUO group, and IRI group. In the gene manipulation experiments, C57BL/6 mice were randomized to different groups underwent different plasmid injection after UUO operation.

Establishment of UUO model was conducted as previously reported^50^. Briefly, the left ureter of C57BL/6 mice was double-ligated with 4-0 silk following a midline incision. The left ureter was only isolated without ligation in the sham group. Mice were ethically sacrificed 7 days after UUO establishment unless indicating a specific time.

For IRI model, C57BL/6 mice were anesthetized with pentobarbital intraperitoneally (i.p.). Both renal pedicles were clamped with microaneurysm clamps for 30 min. During the whole procedure, the core body temperature was kept at 37 °C with an electronic heating pad. After removing the clamps, reperfusion of kidney was visually confirmed. Mice were euthanized 4 weeks after reperfusion.

For ADR nephropathy model, a single dose of ADR, purchased from Santa Cruz biotechnology, Inc (Santa Cruz, USA), was injected through the tail vein at a dose of 9.8 mg/kg to BALB/c mice^51^. Mice were euthanized 4 weeks after ADR injection.

To regulate the expression of Snai1 and p53 in established UUO mice, mRNA encoding either Snai1 or p53 were ligated to a pEX3 vector. shRNA targeting either *Snai1* or *Trp53* were also designed and ligated to a pEX3 vector. All plasmids were synthesized by Genepharma (Shanghai, China). Sequence of mRNA and shRNA could be found in Supplementary Table 1. Plasmid was injected through the tail vein in a hydrodynamic gene delivery method^16,22^ 1 day after UUO establishment.

Bay11-7082 was purchased from MCE (New Jersey, USA) and injected at a dosage of 20 mg/kg i.p. after injection of either Snai1 or p53 overexpression plasmid.

### Histology and immunohistochemistry

H&E staining, Masson trichrome, and Sirius red staining were performed on paraffin sections of 4 μm thickness, as previously described^50^. H&E staining was assessed at ×200 manifestations with tubular injury scores according to the evaluation standard described previously^30^. Tubular injury score was evaluated by two independent pathology doctors without knowing grouping. Positive area of Masson trichrome (blue) and Sirius red (red) staining were evaluated at ×200 manifestations using ImageJ (version 1.52a; National Institutes of Health, Bethesda, MD) to determine ECM deposition.

Immunohistochemistry was performed on paraffin sections of 4 μm thickness according to previous report^30^. Primary antibodies of α-SMA, p53, and p21 were purchased from Abcam (Cambridge, UK). Primary antibodies of F4/80 and Ki-67 were purchased from Cell Signaling Technology (Danvers, MA). Positive area of immunohistochemistry staining was calculated at ×200 manifestations with ImageJ (version 1.52a; National Institutes of Health, Bethesda, MD).

### Western blotting analysis

Protein was extracted from kidney tissue as previously reported^52^. In brief, 15 μg protein of each sample underwent 10% SDS–PAGE electrophoresis and was then transferred to a PVDF membrane. The membrane was blocked with 1% BSA before incubated with primary antibodies at 4 °C overnight. Primary antibodies of E-cadherin, Snai1, and GAPDH were purchased from Cell Signaling Technology (Danvers, MA). Primary antibodies of fibronectin, collagen IV, collagen I, p53, p21, and α-SMA were purchased from Abcam (Cambridge, UK). ImageJ (version 1.52a; National Institutes of Health, Bethesda, MD) was used to quantify blot images. Expression level of target proteins was normalized to that of GAPDH expression level.

### RNA extraction and quantitative PCR

Total RNA was extracted from kidney cortex with TRIzol (Invitrogen, Carlsbad, USA). Reverse transcription and qPCR were proceeded as previous report^21^. The mRNA level of target genes was normalized to that of GAPDH mRNA level and calculated with the 2^−▴▴CT^ method. The primers for target genes can be found in the Supplementary Table 2.

### Single cell isolation and cytometry flow

Single cell was isolated and suspended from kidney cortex tissue as previously reported^10^, and fixed in 1% paraformaldehyde at 4 °C overnight. Single cell suspension was then incubated with propidium iodide/RNase staining solution (Thermo Fisher Scientific, Massachusetts, USA) for 20 min after washing and proceeded for flow cytometry. Cell cycle distribution was analyzed with FlowJo.

### Statistical analysis

Data were presented as mean ± SD. Student’s *t* test or one-way ANOVA analysis were performed for analysis using GraphPad Prism 8.0 Software (GraphPad Software Inc., San Diego, CA, USA). Variance has been taken into account during statistical analysis. Pearson correlation analysis was performed to evaluate the correlation between α-SMA, p53, and p21 immunohistochemistry staining with kidney function in kidney allografts.

## Supplementary information

Figure S1

Figure S2

Figure S3

Figure S4

Figure S5

Figure S6

Figure S7

Supplementary Figure legends

Supplementary Table 1

Supplementary Table 2

## Data Availability

All data supporting this research has been included in this manuscript.
