# Supplementary figures and images for: Snai1-induced partial epithelial–mesenchymal transition orchestrates p53–p21-mediated G2/M arrest in the progression of renal fibrosis via NF-κB-mediated inflammation
Source: Cell Death Dis. 2021 Jan 5;12(1):44. doi: 10.1038/s41419-020-03322-y (PMC7790819; doi:10.1038/s41419-020-03322-y)

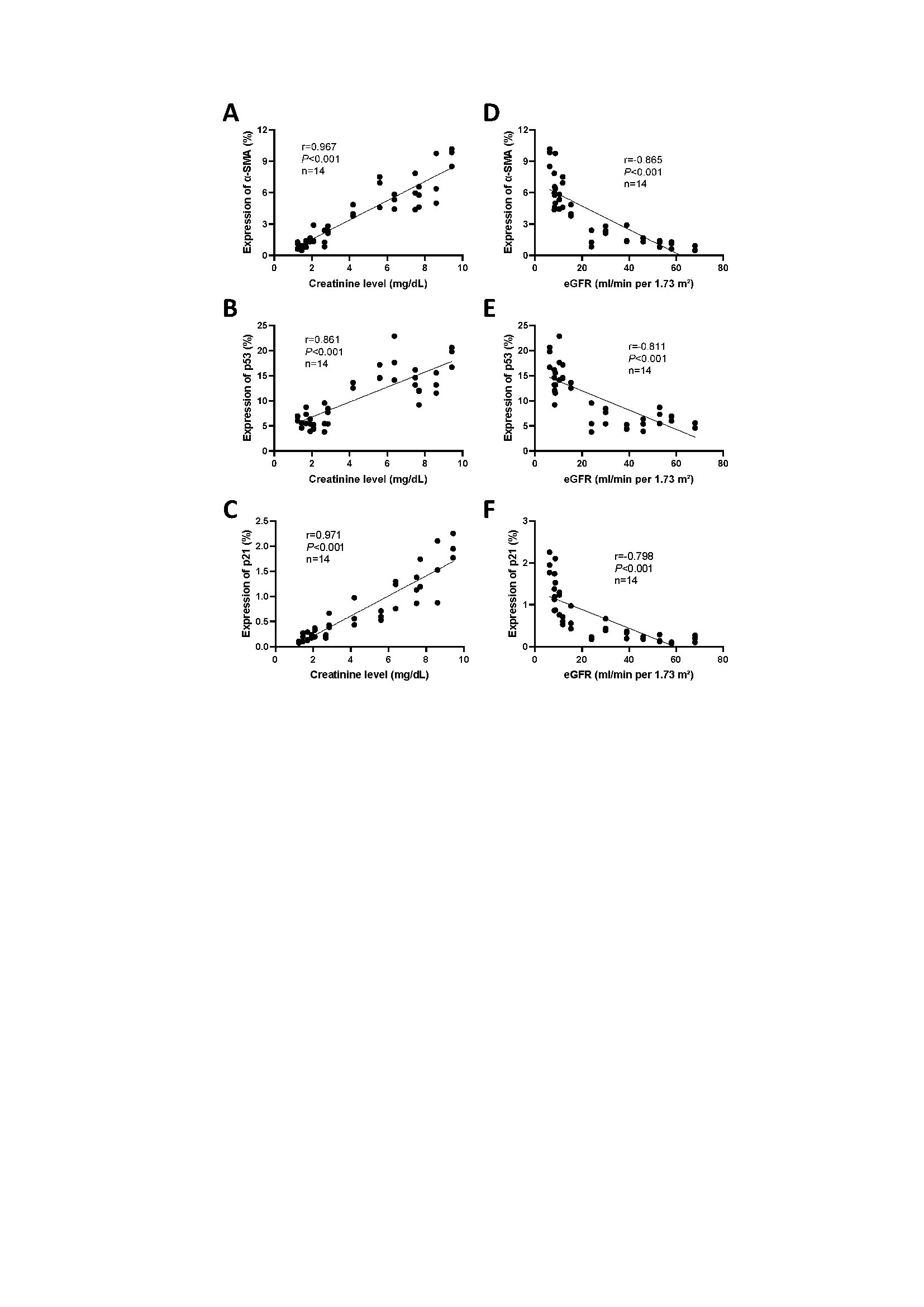

Supplement: Supplementary file 1 — Figure S1 [file 41419_2020_3322_MOESM1_ESM.png]

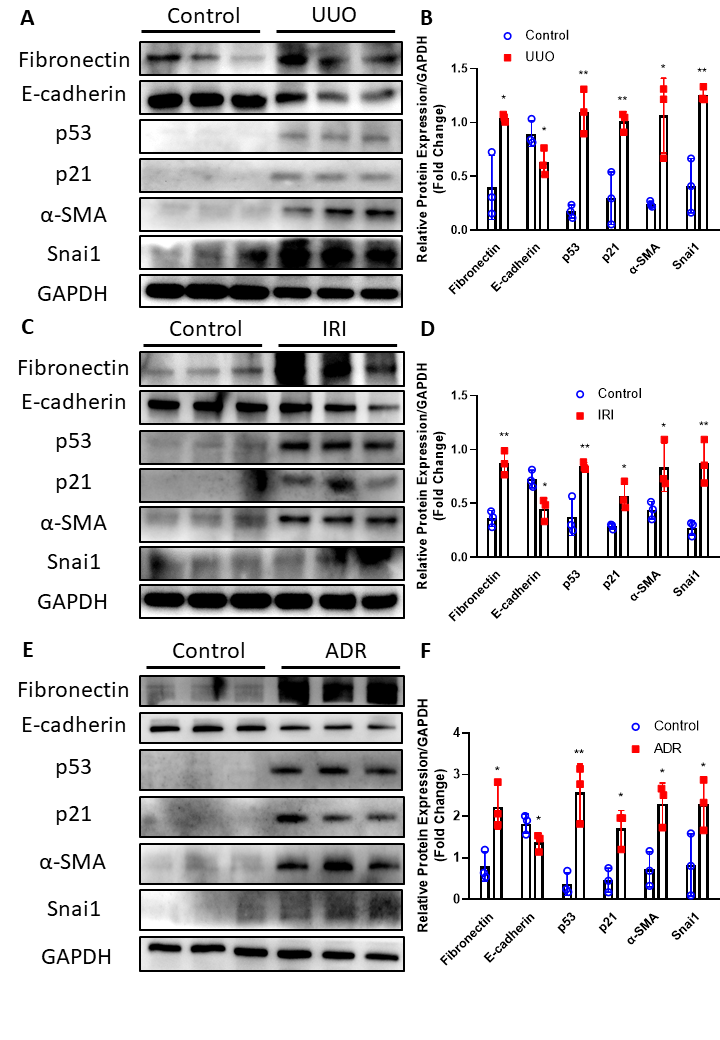

Supplement: Supplementary file 2 — Figure S2 [file 41419_2020_3322_MOESM2_ESM.png]

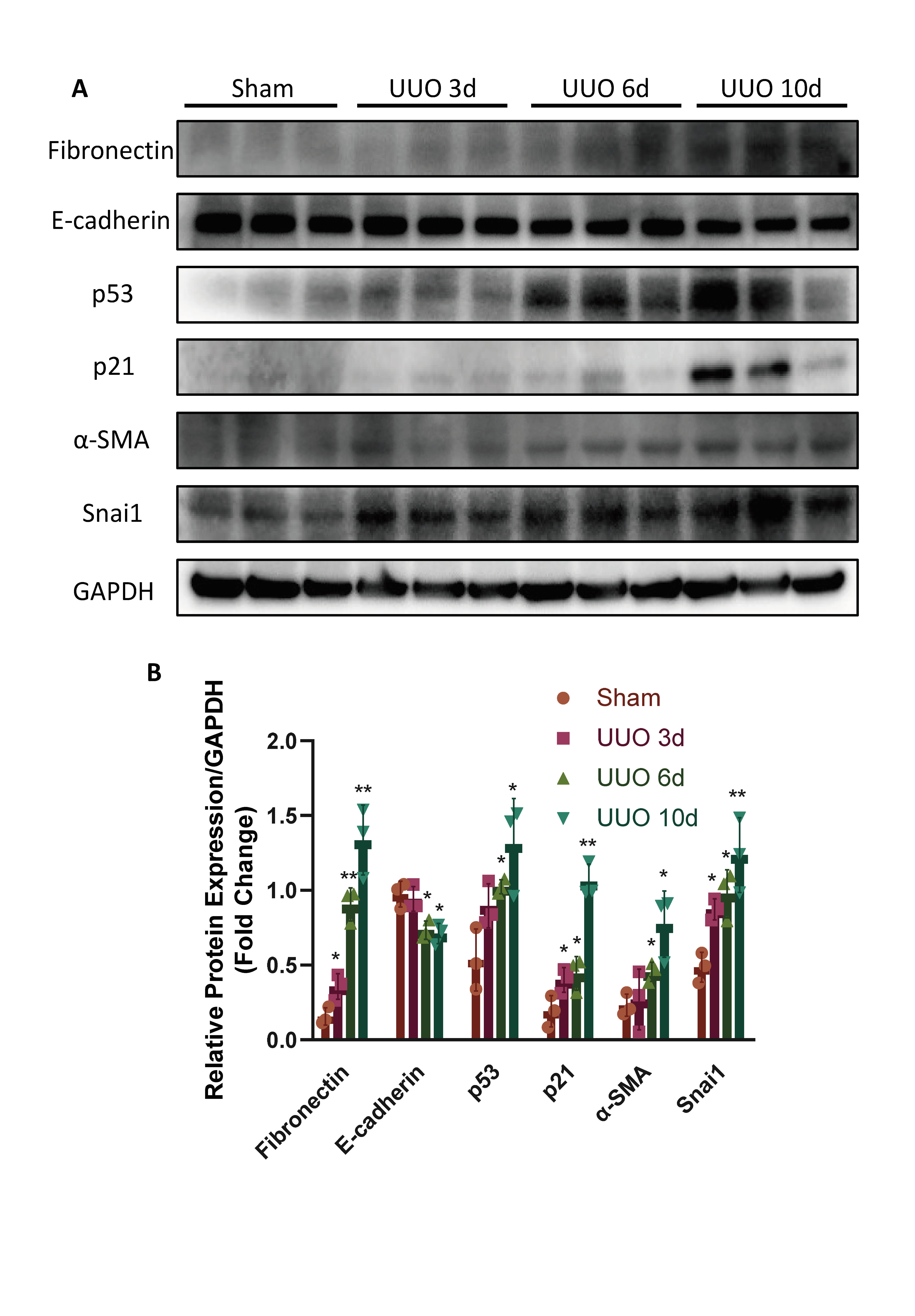

Supplement: Supplementary file 3 — Figure S3 [file 41419_2020_3322_MOESM3_ESM.png]

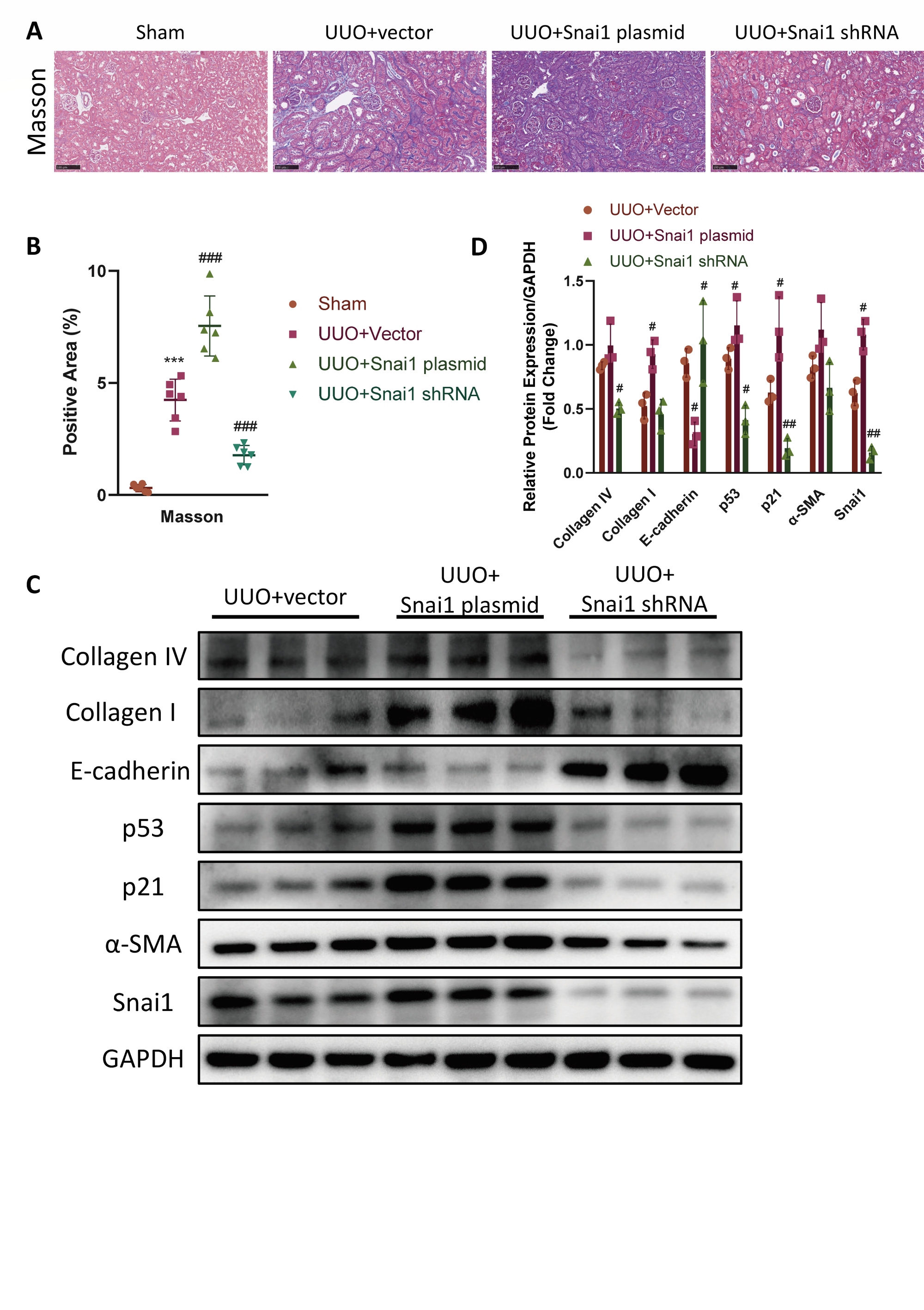

Supplement: Supplementary file 4 — Figure S4 [file 41419_2020_3322_MOESM4_ESM.png]

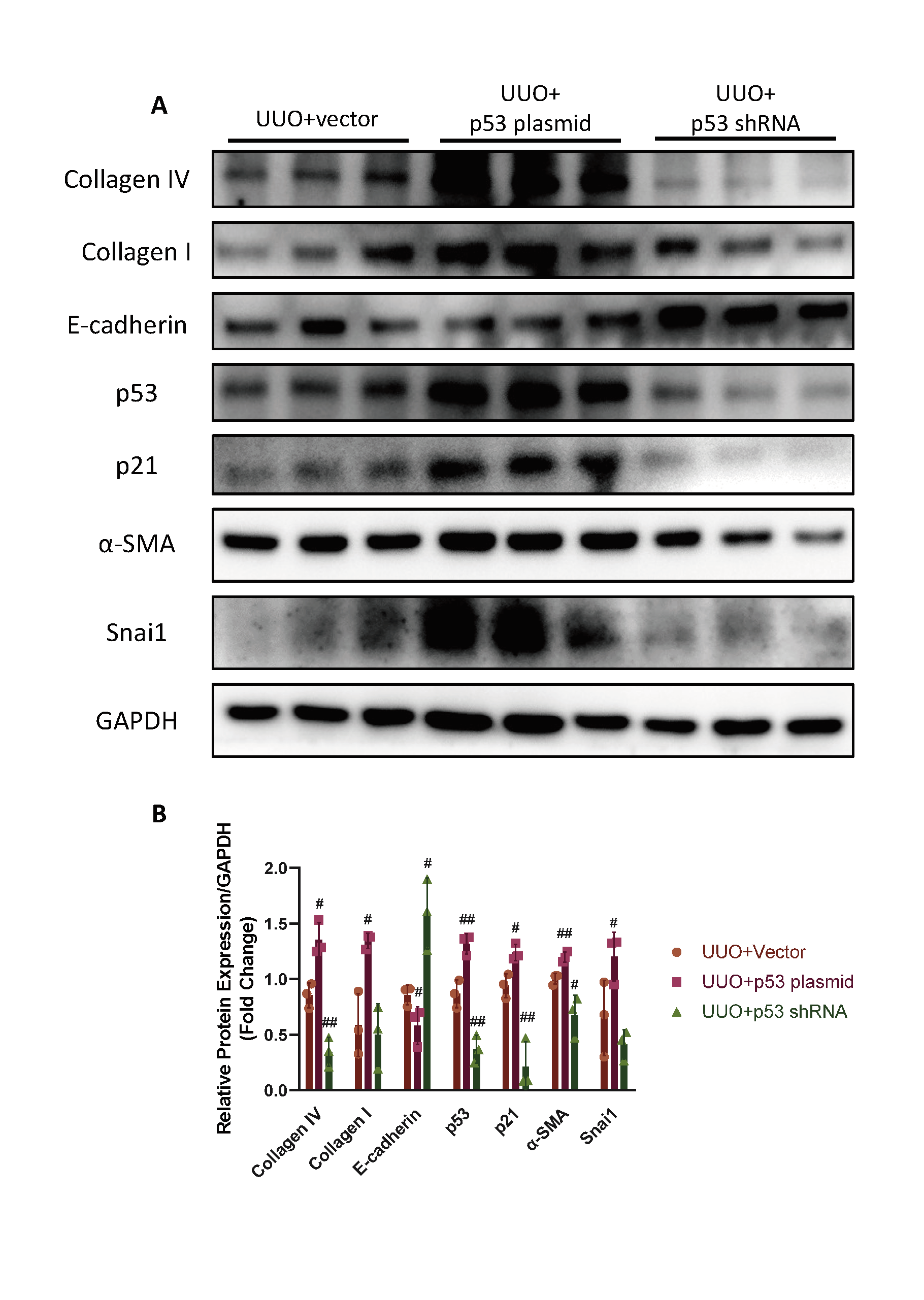

Supplement: Supplementary file 5 — Figure S5 [file 41419_2020_3322_MOESM5_ESM.png]

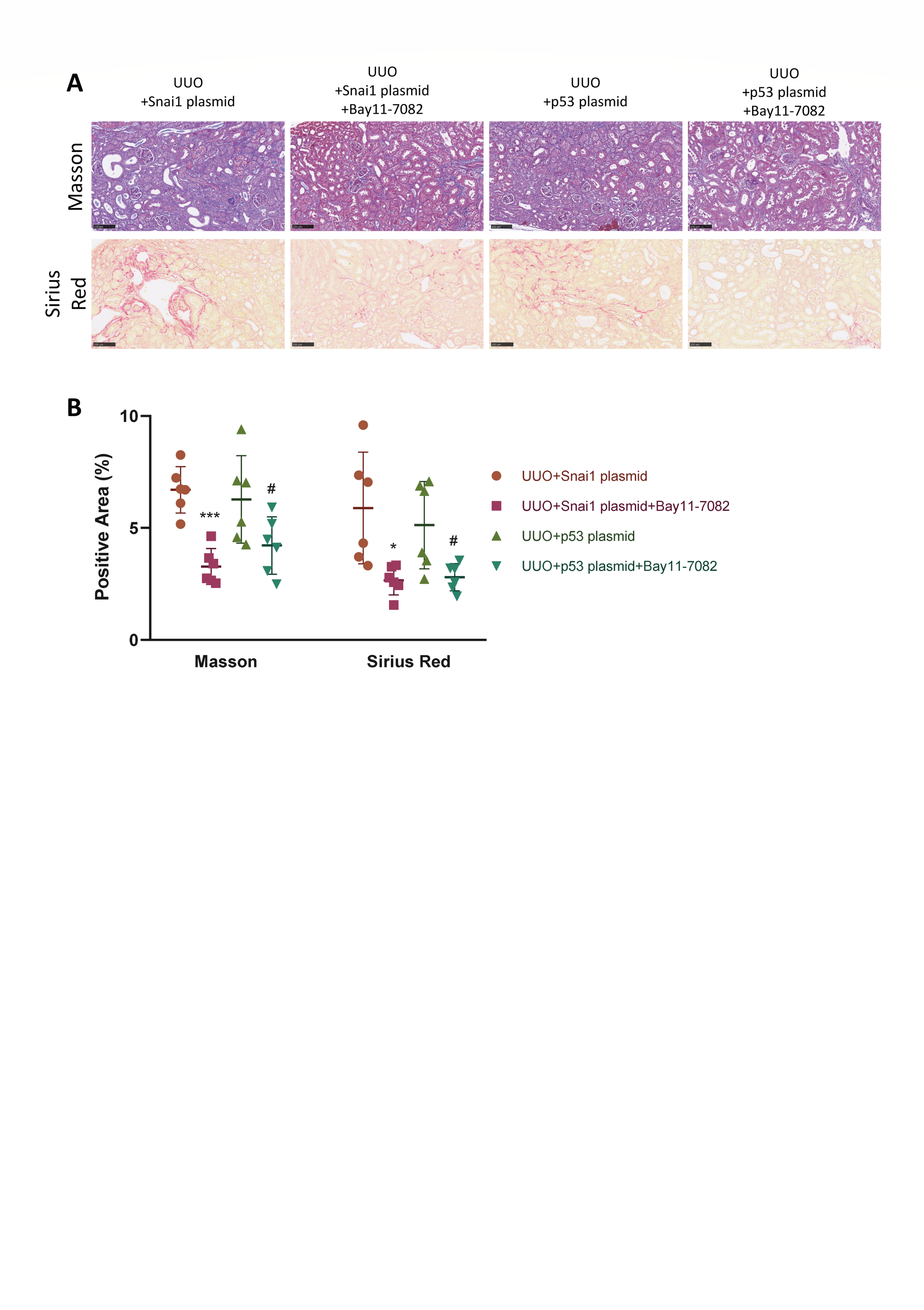

Supplement: Supplementary file 6 — Figure S6 [file 41419_2020_3322_MOESM6_ESM.png]

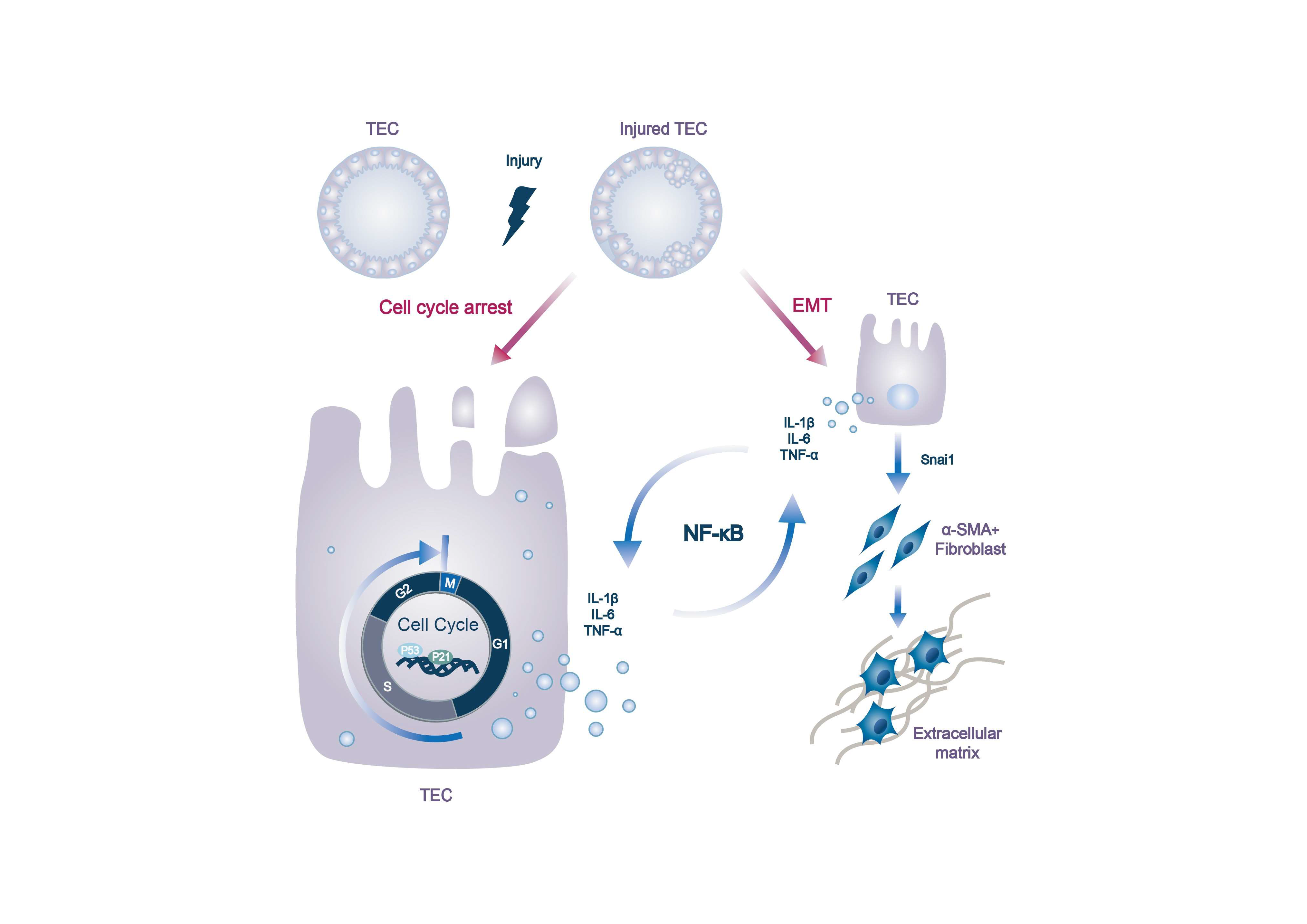

Supplement: Supplementary file 7 — Figure S7 [file 41419_2020_3322_MOESM7_ESM.png]
