# Supplementary material for: Snai1-induced partial epithelial–mesenchymal transition orchestrates p53–p21-mediated G2/M arrest in the progression of renal fibrosis via NF-κB-mediated inflammation
Source: Cell Death Dis. 2021 Jan 5;12(1):44. doi: 10.1038/s41419-020-03322-y (PMC7790819; doi:10.1038/s41419-020-03322-y)
Supplement: Supplementary file 8 — Supplementary Figure legends [file 41419_2020_3322_MOESM8_ESM.docx]

Figure S1. **Expression of α-SMA and p53-p21 axis is correlated with allograft function.** Expression of (A) α-SMA, (B) p53 and (C) p21 is in positive correlation with the creatinine level in kidney recipients. Expression of (D) α-SMA, (E) p53 and (F) p21 is in negative correlation with the eGFR level in kidney recipients.

Figure S2. **EMT and p53-p21 axis were induced in UUO, IRI and ADR nephropathy models.** (A) Western blot analysis of the expression of Fibronectin, E-cadherin, p53, p21, α-SMA and Snai1 in UUO kidneys and (B) their quantifications. (C) Expression of Fibronectin, E-cadherin, p53, p21, α-SMA and Snai1 in IRI kidneys and (D) their quantifications. (E) Western blot analysis of the expression of Fibronectin, E-cadherin, p53, p21, α-SMA and Snai1 in ADR nephropathy kidneys and (F) their quantifications. Western blot analysis of the expression of protein level was normalized to that of GAPDH expression level. * *P*<0.05; ***P*<0.01.

Figure S3. **EMT and p53-p21 axis were upregulated in a time-dependent manner in UUO model.** (A) Western blot analysis of the expression of Fibronectin, E-cadherin, p53, p21, α-SMA and Snai1 in UUO model at different time points, and (B) their quantifications. Western blot analysis of the expression of protein level was normalized to that of GAPDH expression level. * *P*<0.05; ***P*<0.01.

Figure S4. **Regulation of Snai1 expression changes EMT, p53-p21 axis expression and renal fibrosis.** (A) Masson trichrome staining of kidneys of sham, UUO+vector, UUO+Snai1 plasmid and UUO+Snai1 shRNA group, and (B) their quantifications. Scale bar = 100 μm. (C) Western blot analysis of the expression of Collagen IV, Collagen I, E-cadherin, p53, p21, α-SMA and Snai1 in different groups and (D) their quantifications. Western blot analysis of the expression of protein level was normalized to that of GAPDH expression level. *** *P*<0.001 compared with sham group. # *P*<0.05; ##*P*<0.01; ###*P*<0.001 compared with the UUO+vector group.

Figure S5. **Regulation of p53 expression changes EMT and renal fibrosis.** (A) Western blot analysis of the expression of Collagen IV, Collagen I, E-cadherin, p53, p21, α-SMA and Snai1 in different groups and (B) their quantifications. Western blot analysis of the expression of protein level was normalized to that of GAPDH expression level. # *P*<0.05; ##*P*<0.01 compared with the UUO+vector group.

Figure S6. **Treatment of Bay11-7082 attenuated renal fibrosis caused by Snai1 or p53 upregulation.** (A) Masson trichrome and Sirius red staining of UUO kidneys in different groups and (B) their quantifications. Scale bar = 100 μm. ****P*<0.001 compared with UUO+Snai1 plasmid group. # *P*<0.05 compared with UUO+p53 plasmid group.

Figure S7. **Schematic figure of how EMT orchestrates cell cycle arrest during renal fibrogenesis.** Sustained injury turns the repair mechanism of TECs into maladaptive. Some TECs are arrested at G2/M cycle, mediated by p53-p21 axis, to repair DNA damage before going into mitosis. However, persistent arrested cell cycle turns on the SASP, manifested with secretion of inflammatory cytokines, which is mediated by NF-κB. On the other hand, some TECs undergoes the EMT process, acquires partial mesenchymal phenotype and contributes to ECM deposition. TECs undergoes EMT also secrets inflammatory cytokines mediated by NF-κB. Moreover, deposition of ECM also aggravates hypoxia in the interstitium thus exacerbates TEC injury and cell cycle arrest. Therefore, Snai1-induced EMT form a reciprocal loop with p53-p21 axis-mediated G2/M arrest, and this loop is bonded by NF-κB mediated inflammatory response.
